# Epidemiological and environmental investigation of the ‘big four’ *Vibrio* species, 1994 to 2021: a Baltic Sea retrospective study

**DOI:** 10.2807/1560-7917.ES.2024.29.32.2400075

**Published:** 2024-08-08

**Authors:** Greta Gyraitė, Marija Kataržytė, Martynas Bučas, Greta Kalvaitienė, Sandra Kube, Daniel PR Herlemann, Christian Pansch, Anders F Andersson, Tarja Pitkanen, Anna-Maria Hokajärvi, Aune Annus-Urmet, Gerhard Hauk, Martin Hippelein, Eglė Lastauskienė, Matthias Labrenz

**Affiliations:** 1 Marine Research Institute, Klaipeda University, Klaipėda, Lithuania; 2 Institute of Bioscience, Vilnius University, Vilnius, Lithuania; 3 Leibniz Institute for Baltic Sea Research Warnemünde (IOW), Rostock, Germany; 4 Estonian University of Life Sciences, Center for Limnology, Tartu, Estonia; 5 Department of Environmental and Marine Biology, Åbo Akademi University, Turku, Finland; 6 Division of Gene Technology, Science for Life Laboratory, School of Biotechnology, KTH Royal Institute of Technology, Solna, Sweden; 7 Department of Health Security, Finnish Institute for Health and Welfare (THL), Helsinki, Finland; 8 Department of Food Hygiene and Environmental Health, Faculty of Veterinary Medicine, University of Helsinki, Helsinki, Finland; 9 Environmental Health Department, Health Board, Republic of Estonia, Tallin, Estonia; 10 State Office for Health and Social Affairs in Mecklenburg-West Pomerania (LAGuS), Rostock, Germany; 11 Institute of Hospital and Environmental Hygiene, Christian-Albrecht University of Kiel, University Medical Center Schleswig-Holstein, Kiel, Germany

**Keywords:** *Vibrio* spp., infection surveillance, monitoring, bathing waters, Baltic Sea Region, climate change, Europe, waterborne infections, bacterial infections, *Vibrio* infections, infection control, surveillance, epidemiology, statistics

## Abstract

**Background:**

The *Vibrio* genus comprises several bacterial species present in the Baltic Sea region (BSR), which are known to cause human infections.

**Aim:**

To provide a comprehensive retrospective analysis of *Vibrio*-induced infections in the BSR from 1994 to 2021, focusing on the ‘big four’ *Vibrio* species – *V. alginolyticus*, *V. cholerae* non-O1/O139, *V. parahaemolyticus* and *V. vulnificus* – in eight European countries (Denmark, Estonia, Finland, Germany, Latvia, Lithuania, Poland and Sweden) bordering the Baltic Sea.

**Methods:**

Our analysis includes data on infections, *Vibrio* species distribution in coastal waters and environmental data received from national health agencies or extracted from scientific literature and online databases. A redundancy analysis was performed to determine the potential impact of several independent variables, such as sea surface temperature, salinity, the number of designated coastal beaches and year, on the *Vibrio* infection rate.

**Results:**

For BSR countries conducting surveillance, we observed an exponential increase in total *Vibrio* infections (n = 1,553) across the region over time. In Sweden and Germany, total numbers of *Vibrio* spp. and infections caused by *V. alginolyticus* and *V. parahaemolyticus* positively correlate with increasing sea surface temperature. Salinity emerged as a critical driver of *Vibrio* spp. distribution and abundance. Furthermore, our proposed statistical model reveals 12 to 20 unreported cases in Lithuania and Poland, respectively, countries with no surveillance.

**Conclusions:**

There are discrepancies in *Vibrio* surveillance and monitoring among countries, emphasising the need for comprehensive monitoring programmes of these pathogens to protect human health, particularly in the context of climate change.

**Key public health message**

**What did you want to address in this study and why?**

*Vibrio* bacteria live in coastal waters, such as the Baltic Sea, which has low-medium salinity and is above 15 °C in the summer months. Vibriosis can be acquired from eating raw seafood or swimming with open wounds, with an elevated risk for immunocompromised people. We aimed to study and predict how *Vibrio* infections might spread in eight countries bordering the Baltic Sea. With the waters warming up, this region could soon become a ‘hotspot’ for these infections.

**What have we learnt from this study?**

We found an increase in recorded vibriosis from 1994 to 2021, along with a widespread presence of *Vibrio* bacteria in Baltic Sea countries that collect data. Our prediction model, based on environmental factors like temperature and salinity, suggests that *Vibrio* infections may be underestimated in countries without official monitoring. This leaves potential health risks to coastal zone visitors unknown.

**What are the implications of your findings for public health?**

Our study highlights the need for an adequate system of infection surveillance and water monitoring in the Baltic Sea region and across Europe. Public reporting systems are crucial to ensure the safety of tourists and coastal communities. Additionally, an international framework is needed to facilitate the exchange of epidemiological, clinical and environmental information.

## Introduction

The *Vibrio* genus comprises more than 100 bacterial species, approximately 12 of which are known to cause human infections referred to as vibriosis. *Vibrio* spp. are omnipresent in warm estuarine (> 15 °C) and low to moderately saline (5–25 practical salinity units (PSU)) coastal waters. Clinical manifestations of vibriosis include mild wounds, ear infections and gastroenteritis, while *Vibrio vulnificus* can cause severe wound infections that can rapidly lead to septicaemia, resulting in a case fatality rate of 50% [1]. The majority of illnesses usually occur through the consumption of raw or undercooked seafood and via skin wounds (small mild lacerations to larger open wounds) contact with seawater or estuary bathing waters, while severe illnesses can be acquired by individuals with underlying conditions such as diabetes, liver diseases or immune disorders, and also elderly (> 65 years) people [2]. The ageing population faces an increased incidence of chronic conditions, thereby amplifying the susceptibility to *Vibrio* infection-related health risks [3].

Notable infectious species present in the Baltic Sea region (BSR) include *Vibrio alginolyticus*, nontoxigenic *Vibrio cholerae* (non-O1/non-O139), *Vibrio parahaemolyticus* and *V. vulnificus,* collectively known as the ‘big four’ [4]. Furthermore, the brackish, fast-warming and organic-rich waters of the BSR provide an ideal environment for the growth and proliferation of *Vibrio* spp. [5,6]. *Vibrio* abundances are notably influenced by temperature in spring, salinity in winter and chlorophyll a in spring and summer [7]. In addition, studies by Baker-Austin et al. [8] and Trinanes and Martinez-Urtaza [9] show that climate change may cause even more vibriosis cases, and that the BSR may become a hotspot for non-cholera vibriosis in the coming decades. This is of concern for public health, but also for the local economy and the European Union’s blue economy [10], as high-quality beaches and bathing waters are important for the tourism industry in the BSR.

Currently, no global systematic *Vibrio* infection surveillance framework exists. Only a few countries including Canada (since 1997), Germany (since 2004), and the United States (US) (since 1988) have established surveillance systems [11] and provide some valuable information on infection dynamics. For example, according to the California Department of Public Health, *Vibrio* spp. cause 80 thousand illnesses, 500 hospitalisations and ca 100 deaths each year in the US [12,13]. Moreover, reported vibriosis increased tenfold from 0.09 cases per 100,000 population in 1996 to 0.9 cases per 100,000 in 2018 [13,14].

Despite *Vibrio* infections being documented within the BSR since 1978 [15], vibriosis remains a non-notifiable disease in Europe, lacking consistent surveillance in bathing waters [1]. In addition, the epidemiological data on *Vibrio* infections lack precision, e.g. the date of infection, which is mostly provided on an annual level, and the place of exposure. Furthermore, there is a need for improved diagnostic detection and clinical awareness [16]. Existing studies analysing the epidemiological data of *Vibrio* in the BSR are fragmented, primarily focusing on individual cases within specific countries or associations with heat waves [8,17-19].

The geographic distribution of different *Vibrio* species within the BSR and how their presence is impacted by environmental conditions such as temperature and salinity remain incompletely defined. Therefore, the objectives of this study were (i) to examine the dynamics of *Vibrio* infections and environmental occurrence in eight BSR countries to date, and provide specific case studies from Estonia, Germany and Sweden, and (ii) to assess the role of environmental factors, specifically salinity and temperature, on several *Vibrio* species using historical data to predict cases in Germany, Lithuania and Poland using multivariate statistical models. 

## Methods

### Study design and setting

This retrospective study covered the period from 1994 to 2021, encompassing all eight European countries along the Baltic Sea coastline: Denmark, Estonia, Finland, Germany, Latvia, Lithuania, Poland and Sweden. Multiple approaches were employed for data gathering. Initially, two distinct surveys were devised to collect (i) epidemiological information from infection case surveillance data and (ii) environmental monitoring data on *Vibrio* spp. See Supplementary Table S1 and Table S2 for examples of surveys. 

The surveys were distributed to the partners of the BiodivERsA project ‘BaltVib’ (https://www.io-warnemuende.de/baltvib-home-en.html). Coordinator: Leibniz Institute for Baltic Sea Research Warnemünde (IOW), Germany. Members: Åbo Akademi University, Finland; Estonian University of Life Sciences (EMÜ), Estonia; Helmholtz-Zentrum für Ozeanforschung (GEOMAR), Germany; Marine Research Institute of Klaipėda University (KU), Lithuania; National Marine Fisheries Research Institute, Poland (NMFRI); Royal Institute of Technology (KTH), Sweden; University of Copenhagen (UCPH), Denmark. 

The ‘BaltVib’ consortium members then conducted surveys with representatives of the respective national or regional public health authorities in each country. In addition, a literature search by the authors of this study was carried out to comprehensively examine cases of infection and environmental prevalence to fill gaps in the dataset where certain countries could not provide data, and to map the geographical distribution of both cases of infection and *Vibrio* occurrence.

### Epidemiological data collection

Surveillance data collection on human vibriosis cases included country, infection date, bathing/infection site and *Vibrio* species. The reporting criteria for Denmark [18], Estonia, Germany and Sweden are similar: a laboratory-confirmed case was defined as an isolation of *Vibrio* spp. other than *V. cholerae* O1/O139 and clinical criteria including otitis, wound infection, gastroenteritis and septicaemia. Cases without a travel history, i.e. cases reported in Estonia, were considered to be non-travel-related because most were confirmed for *V. cholerae* non-O1/O139 during the summer months (end of May–August), when *V. cholerae* non-O1/O139 was found in all bathing sites (n = 15) and years (2020–21) tested, and all affected people live in the coastal counties. In Finland, a case was defined as *V. cholerae* including non-O1/O139. The place of residence was used as a proxy when the place of infection was not reported. More details about the infection surveillance can be found in Supplementary Table S3.

### Environmental data collection

Data collected on *Vibrio* monitoring included country, date, monitoring site, *Vibrio* spp. quantity or presence, sea surface temperature (SST) and sea surface salinity (SSS) data. The SST and SSS records were sourced from literature, governmental sources and institutional data, either directly or from official online databases. The details about *Vibrio* spp. monitoring in bathing waters are provided in Supplementary Table S4. The number of designated coastal beaches at the nomenclature of territorial units for statistics (NUTS) 2 and/or 3 levels was counted from the European Environment Agency’s interactive map in 2022 [20]. *Vibrio* spp. presence indicated as ‘Found’ and absence indicated as ‘Not found’ across the BSR coastline were mapped using QGIS software (version 3.36.3).

### Statistical analysis

Before the analysis, the normality of variables (the number of infections among the counties and the years) was tested using the Kolmogorov–Smirnov test. The data deviated from the normal probability distribution. Therefore, the non-parametric Kruskal–Wallis test was applied to compare the spatial and temporal differences. ﻿To investigate the relationships between the number of *Vibrio* spp. infections and abiotic parameters, the Spearman correlation (r_s_) was used. The significance level (α) used was 5%. Descriptive statistics are shown as mean ± standard deviation for continuous data. The statistical analyses were conducted using the R software (version 4.3.2).

The potential impact of several independent variables (predictors) on the infection rates of *Vibrio* was investigated with a redundancy analysis (RDA). Specifically, the influence of parameters such as SST, SSS, the number of designated coastal beaches and year was examined. The multicollinearity among predictors was assessed by the variance inflation factor, which was lower than 3. The RDA model was calibrated with the infection data from Sweden, as they covered the broadest range of salinity (2–23 PSU) in the BSR. The data included the total *Vibrio* infections and specific species (*V. alginolyticus*, *V. cholerae*, *V. parahaemolyticus* and *V. vulnificus*) at the NUTS level 3 (county) for a specific period (2014–21). The RDA model built on these data was then internally (using the Swedish data) and externally (using the German data NUTS level 2 covering the period of 2014–21) validated and subsequently used for *Vibrio* infection predictions in Germany, Poland and Lithuania (period of 2014–21). Latvia is not included in the projections because of a lack of measurements of environmental parameters. The mean absolute error (MAE) was calculated to evaluate the accuracy of both validations.

The importance of the predictors in the RDA model was assessed using the marginal significance (F) of the explanatory variables (by the Monte Carlo permutation test with 999 permutations) and the variance partitioning based on the adjusted R^2^. For the graphical representation of the linear relationships between the response and predictors, we opted for an RDA triplot and biplots with a scaling factor of 2 [21]. Moreover, to interpret the real values of predictors in the multivariate dimension of RDA, we superimposed smooth surfaces of predictors onto the RDA biplots. This was achieved by using thin plate splines, employing the generalised additive model approach with a cross-validatory selection of smoothness. The RDA was carried out using the ‘vegan’ package [22].

## Results

This retrospective study examined the epidemiology of *Vibrio* infections and environmental occurrence data of *Vibrio* spp. in eight European countries along the Baltic Sea coastline. In the BSR, five of the eight countries (Denmark, Estonia, Finland, Germany and Sweden) maintain national surveillance systems for cases of vibriosis, and four (Estonia, Finland, Germany and Sweden) partially provided data. Denmark did not provide data because of confidentiality concerns. Three countries, Latvia, Lithuania and Poland, have not yet implemented surveillance systems or monitoring programmes explicitly targeting *Vibrio* bacteria (except for *V. cholerae* O1/O139); thus, no data could be collected.

### Epidemiological and environmental investigation

In total, we obtained data on 1,553 *Vibrio* cases of vibriosis, of which 691 cases came from official data provided by designated national public health authorities from three countries (Estonia, Germany and Sweden) (Table). The highest number of infections was reported in Sweden (n = 625). Germany reported 57 infections, while Estonia reported 9 infections. The data concerning *Vibrio* infections in Finland (n = 216), Denmark (n = 638) and Poland (n = 8) were obtained from scientific publications, comprising a notable portion of the overall dataset. Specifically, these three countries contribute to 55.5% of the total number of infections, representing a substantial portion of the observed cases (n = 862). 

**Table:** Reported cases according to *Vibrio* species, Baltic Sea region, 1994–2021 (n = 1,553)

Country	Period	*Vibrio* species (n)						Sources
		Total cases	Va	Vc	Vp	Vv	*Vibrio* spp.^a^	
Denmark	2010–18	638	333	18	156	32	99	[19]
Estonia	2020–21	9	0	7	0	0	2	Public health agency of Estonia
Finland	2005–14	216	0	45	0	0	141	[17,18]
	2018			26	3	1	0	
Germany	1994–21	57	2	3	3	28	21	Public health agency of Germany
Latvia	No surveillance performed							
Lithuania	No surveillance performed							
Poland	2018–21	8	0	6	0	2	0	[18,38]
Sweden	2004–21	625	72	183	59	22	289	Public health agency of Sweden
**Total**		1,553	407	288	221	85	552	NA

NA: not applicable; Va: *Vibrio alginolyticus*; Vc: *Vibrio cholerae* non-O1/O139; Vp: *Vibrio parahaemolyticus*; Vv: *Vibrio vulnificus*.

^a^ *Vibrio* spp. indicate unspecified *Vibrio* species.

In addition to the 216 vibriosis cases identified in Finland through scientific literature [17,18], 221 cases of *V. cholerae* were recorded by the Finnish Institute for Health and Welfare between 1995 and 2021. Over the years, the number of infections in Finland varied from 1 in 2000, 2008 and 2009 to 43 infections registered in 2014. An overview of cases of *V. cholerae* reported in Finland is provided in Supplementary Figure S1. For all 221 cases, neither the exact date of infection nor the transmission pathways are known. Therefore, this dataset was not included in further analyses.

**Figure 1:**
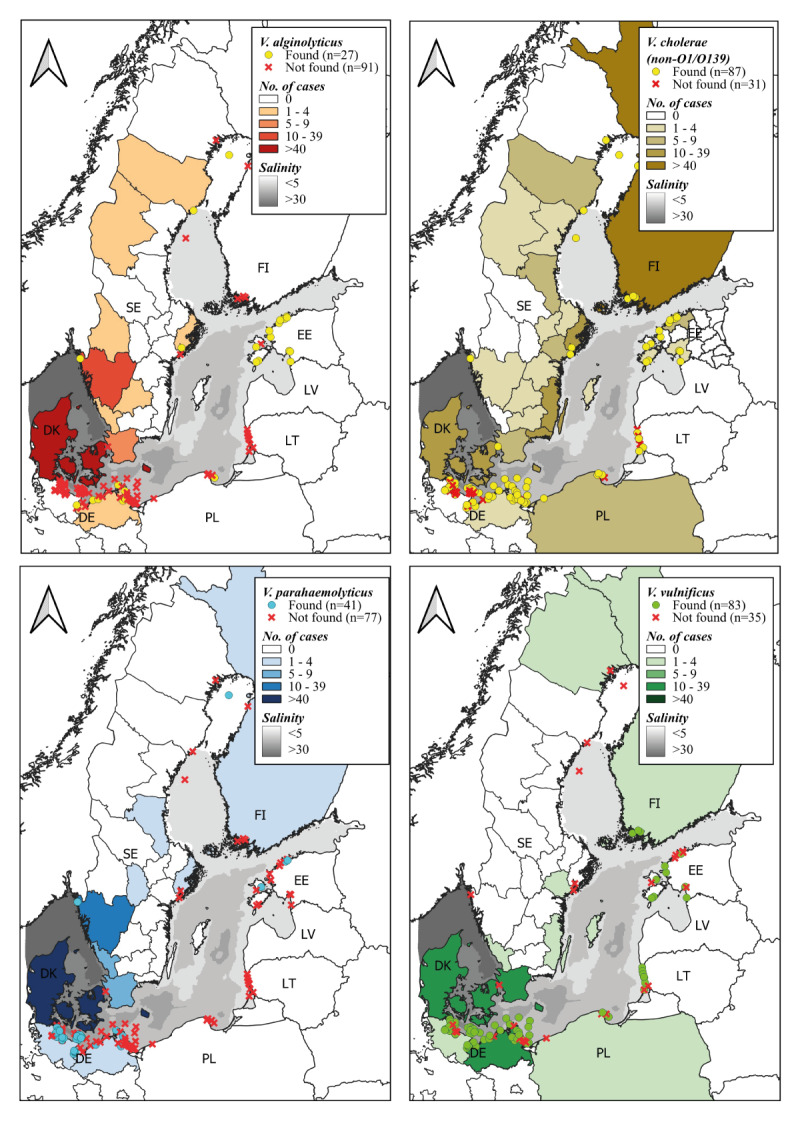
*Vibrio* species distribution in coastal waters and number of cases, Baltic Sea region, 1994–2021 (n = 7 countries)

DE: Germany; DK: Denmark; EE: Estonia; FI: Finland; LV: Latvia; LT: Lithuania; PL: Poland; SE: Sweden.

From 1994 to 2021, infections caused by *Vibrio* bacteria in Germany, Sweden and Estonia were predominantly observed from May to November, coinciding with SST ranging from 13.4 to 25.7 °C and SSS levels ranging from 2 to 22.1 PSU (Figure 1). However, annual mean SST across the BSR did not differ significantly (p < 0.05). Notably, the highest incidence of total *Vibrio* bacterial infections was recorded in 2018, a year characterised by exceptionally elevated SST during the summer months. The Spearman correlation analysis associated a significant negative relationship between the total number of infections (n = 691) and salinity (r_S_ = −0.5, p < 0.05) but no significant relationship with temperature.

Data are based on official infection surveillance data and published literature and water monitoring data. No data were reported by Latvia. Details of data sources are provided in Supplementary Table S3 and Table S4.

A detailed examination of the official water monitoring records (Estonia, Finland and Germany) and records from published scientific literature (Lithuania, Poland and Sweden) revealed distinct distribution patterns of various *Vibrio* spp. within specific SST and SSS ranges in the BSR (Figure 1). We analysed only data of the most common 'big four' *Vibrio* species in more detail (n = 2,011).

**Figure 2:**
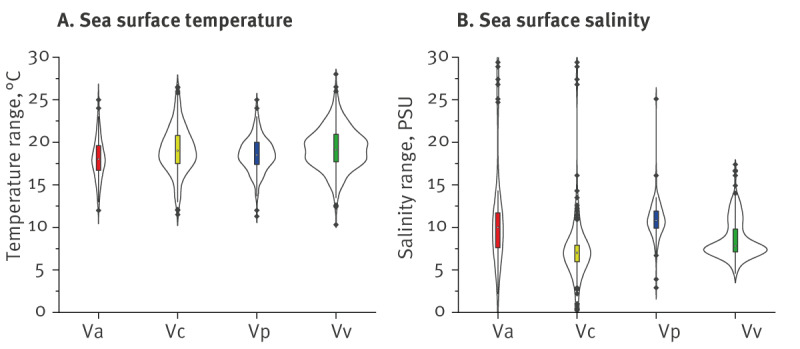
Sea surface temperature and salinity ranges of *Vibrio* species prevalence, Baltic Sea region, 2004–2021 (n = 2,011 from 5 countries)

PSU: practical salinity unit; Va: *V. alginolyticus*, Vc: *V. cholerae* non-O1/O139, Vp: *V. parahaemolyticus*; Vv: *V. vulnificus.*

*Vibrio alginolyticus* exhibited growth within the temperature range of 12 to 25 °C across a salinity range of 2.2 to 29.4 PSU (Figure 2). Of the total, 26.2% (n = 407) of all infections in the BSR were caused by *V. alginolyticus*. In Denmark, from 2010 to 2018, 333 cases of *V. alginolyticus* cases of vibriosis were documented, accounting for 52.2% of all reported infections in the country (Table; Figure 1). *Vibrio cholerae* non-O1/O139 exhibited a wider distribution and was found across the SST range of 11.5 to 26.5 °C, displaying a consistent distribution pattern following the Baltic Sea's salinity gradient from 0.24 to 29.4 PSU. Of the ‘big four’, it was the dominant *Vibrio* species found in the BSR. Non-toxigenic *V. cholerae* caused 18.5% (n = 288) of all cases. *Vibrio parahaemolyticus* was detected in temperatures ranging from 11.3 to 25.0 °C, with higher average salinity conditions of 10.6 ± 2.3 PSU, observed primarily in Germany and some locations in Estonia; *V. parahaemolyticus* caused 14.2% (n = 221) of all vibriosis cases. In the BSR waters, *V. vulnificus* was detected within the temperature range of 13.5 to 28.0 °C and was notably absent from regions characterised by lower than 5 PSU salinity levels, such as the Gulf of Bothnia (Figures 1 and 2) and, unlike the rest of the *Vibrio* species, was not present in waters with higher than 17 PSU. In the BSR *V. vulnificus* was the least frequently reported species, accounting for 5.5% (n = 85) of the cases.

Data are based on official monitoring of water data (Germany, Estonia, Finland) and published literature (Sweden, Lithuania). Details of data sources are provided in Supplementary Table S4.

### *Vibrio* cases and occurrence: official data from three countries

#### Estonia

The infection surveillance in Estonia was initiated in 2020. In 2 years (2020–21), the Public Health Agency of Estonia documented a total of nine vibriosis cases. Specifically, four in 2020 and five in 2021. Seven of these cases were reported in Harju County, on the southern coast of the Gulf of Finland, and one each in Saare County on Estonia's largest island and Pärnu County on the coast of the Gulf of Riga. Notably, seven of nine cases were attributed to *V. cholerae* (non-O1/O139), while the remaining two were not ascribed to a particular *Vibrio* spp.

Official *Vibrio* spp. monitoring data in the coastal bathing waters of Estonia cover a comparably short period from 2019 to 2021. However, various *Vibrio* species were found: *V. alginolyticus*, *V. aesturianus*, *V. anguillarum*, *V. cincinnatiensis*, *V. cholerae* (non-O1/O139), *V. diazotrophicus*, *V. fluvialis*, *V. furnissi*, *V. navarrensis*, *V. harveyi*, *V. mentschinkowi*, *V. vulnificus* and *V. parahaemolyticus*.

The non-O1/O139 strain of *V. cholerae* was found in all tested bathing sites and years along the Estonian coast of the Baltic Sea, and it was the dominant *Vibrio* species found in all bathing sites. *Vibrio cholerae* (non-O1/O139) was observed at the range of the SST of 11.5–26 °C. *Vibrio vulnificus* was observed at the range of the SST of 13.5–28.0 °C (average suitable temperature: 19.6 ± 3.9 °C) and was found in eight of 15 tested bathing sites along the Estonian coast of the Baltic Sea. *Vibrio parahaemolyticus* was observed at the range of the SST of 13.9–17.6 °C (average suitable temperature: 16.1 ± 1.9 °C). It was found only once in three of 15 tested bathing sites along the Estonian coast of the Baltic Sea. *Vibrio alginolyticus* was observed at the range of the SST of 12–25 °C (average suitable temperature: 18.1 ± 2.8 °C) and was the second most common *Vibrio* species found in 14 of 15 tested bathing sites along the Estonian coast of the Baltic Sea. The geographical distribution of ‘big four’ *Vibrio* species along the Estonian Baltic Sea coast can be found in Supplementary Figure S2.

#### Germany

In the period 1994 to 2021, in total, 57 cases have been reported by the Public Health Agencies of Schleswig-Holstein and Mecklenburg-Vorpommern, German federal states with Baltic Sea coastline. Eight infections occurred in Schleswig-Holstein between 2014 and 2021, and 49 in the Mecklenburg-Vorpommern between 1994 and 2018. *V. alginolyticus* was responsible for two infections, and non-toxigenic *V. cholerae* and *V. parahaemolyticus* were responsible for three infections each. The highest number of infections was caused by *V. vulnificus* (n = 28). For 21 cases, specific *Vibrio* species were not determined. Over the years, there was a significant positive correlation between infection and water salinity (r_S_ = 0.56, p < 0.05), while the correlation with temperature records was not significant.

*Vibrio* spp. were monitored in 2015, 2016 and 2018 in various bathing sites along the Baltic Sea coast of the Schleswig-Holstein. *Vibrio cholerae* (non-O1/O139) was observed at an SST range of 13–24 °C (average suitable temperature: 18.5 ± 2.1 °C) and was found at 17 of 32 bathing sites tested on the coast of Schleswig-Holstein. *Vibrio parahaemolyticus* and *V. vulnificus* were observed at 30 and 25 tested bathing sites, respectively, at an SST range of 13–24 °C (average suitable temperature: 18.5 ± 2.2 °C). The abundance of all three species *V. cholerae* (r_S_ = 0.14, p < 0.05), *V. parahaemolyticus* (r_S_ = 0.21, p < 0.05) and *V. vulnificus* (r_S_ = 0.42, p < 0.05) were significantly positively correlated with SST (n = 272).

The monitoring of *Vibrio* spp. presence at the coast of Mecklenburg-Vorpommern included data from 2004 to 2021, taken at various bathing sites. During monitoring, different *Vibrio* species were found: *V. alginolyticus*, *V. cholerae*, *V. metschnikovii*, *V. parahaemolyticus*, *V. vulnificus* and *V. fluvialis*. Quantification of the abundance of *V. vulnificus* took place from 2008 to 2021. *Vibrio vulnificus* abundance and SST differed significantly among five analysed bathing sites (with sufficient amount of data) and years (p < 0.05). Meanwhile, salinity differed among bathing sites but did not differ among years. Two bathing sites – Karlshagen and Lubmin – are associated with the reporting of vibriosis cases. In Karlshagen, *V. vulnificus* was observed at the range of the SST of 11.3–23.2 °C (average suitable temperature: 18.1 ± 2.3 °C) and at the range of SSS of 4.7–7.8 PSU (average suitable salinity: 6.9 ± 0.6 PSU). In Lubmin, *V. vulnificus* was observed at the range of the SST of 12.3–26 °C (average suitable temperature: 18.9 ± 2.7 °C) and at the range of SSS of 4.2–8.2 PSU (average suitable salinity: 6.9 ± 0.9 PSU). In each of those sites, three cases were reported, and a significant positive relationship (r_S_ = 0.36; p < 0.05) was observed between temperature and *V. vulnificus* abundance in most probable number per litre (MPN/L). Supplementary Figure S3 provides the temporal distribution of *V. vulnificus* in two Mecklenburg-Vorpommern bathing sites, Karlshagen and Lubmin, in 2008–21.

#### Sweden

**Figure 3:**
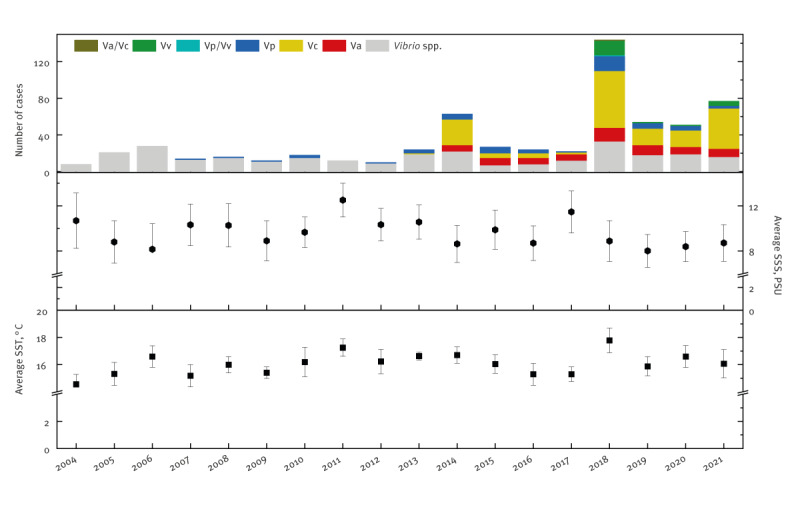
Temporal distribution of *Vibrio* spp. infections, average temperature (June–September) and salinity reported by 14 counties with a Baltic Sea coastline, Sweden, 2004–2021 (n = 550)

PSU: practical salinity unit; SSS: sea surface salinity; SST: sea surface temperature; Va: *V. alginolyticus;* Vc: *V. cholerae* non-O1/O139; Vp: *V. parahaemolyticus*; Vv: *V. vulnificus*.

From 2004 to 2021, 625 cases of vibriosis have been reported by the Public Health Agency of Sweden. Over the years, the total number of cases varied from 8 reported in 2004 to 144 in 2018, and numbers grew exponentially (R^2^ = 0.43) over time (Figure 3). For all 625 cases, neither the exact date of infection nor the specific bathing site where the infection might have occurred was known. All cases were reported at the NUTS 3 level. 

Data are based on official surveillance of vibriosis cases (reporting counties Blekinge, Gävleborg, Gotland, Halland, Kalmar, Norrbotten, Östergötland, Skåne, Södermanland, Stockholm, Uppsala, Västerbotten, Västernorrland, Västra Götaland) and environmental data extracted from the database (SHARKweb), hosted by the Swedish Meteorological and Hydrological Institute. The grey bars indicate detections identified to the genus level. More details on sources of data can be found in Supplementary Table S3. In some cases, two *Vibrio* species were identified at the same time (Va/Vc and Vp/Vv).

**Figure 4:**
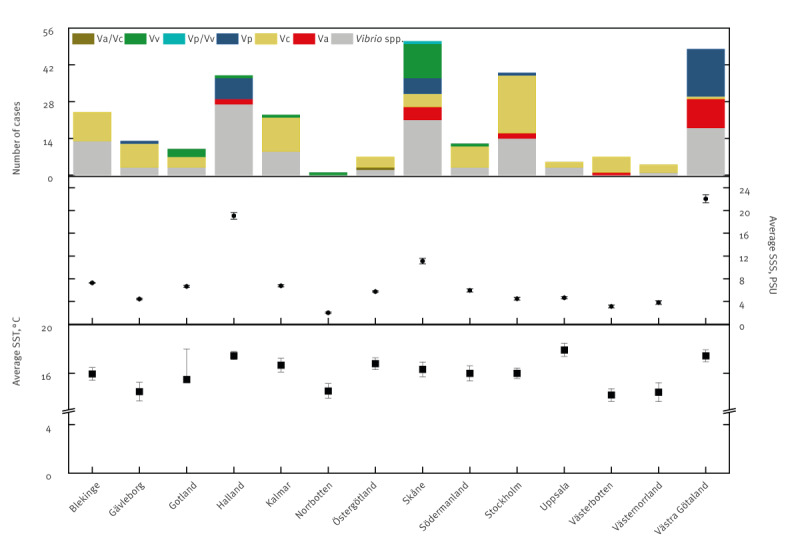
Geographical distribution of *Vibrio* species cases and average temperature (June−September) and salinity reported by 14 counties with a Baltic Sea coastline, Sweden, 2004–2021 (n = 550)

PSU: practical salinity unit; SSS: sea surface salinity; SST: sea surface temperature; Va: *V. alginolyticus;* Vc: *V. cholerae* non-O1/O139; Vp: *V. parahaemolyticus*; Vv: *V. vulnificus*.

The number of reported cases differed among the counties and the years (p < 0.05). The highest number of cases over the studied period were reported by Skåne county (n = 144), followed by Västra Götaland (n = 106) and Stockholm (n = 101) (Figure 4). No cases were reported by Dalarna County, which is inland and has no coastline. In total, 75 infection cases were reported in the Swedish counties that are not located at the Baltic Sea coast (6/21 total counties). Therefore, linking those infection cases with the environmental conditions is not possible, and they were disregarded from further analyses.

Data are based on official surveillance of vibriosis cases and environmental data such as temperature and salinity (June–September) extracted from the database (SHARKweb), hosted by the Swedish Meteorological and Hydrological Institute. The grey bars indicate detections identified to the genus level. More details on sources of data can be found in Supplementary Table S3.

From 2004 until 2013, *V. parahaemolyticus* was the only species specifically identified as the causative agent of vibriosis in Sweden, causing 59 cases (Figure 3). Infections by *V. parahaemolyticus* were recorded in higher numbers in the counties bordering coastal waters with higher salinity, such as Västra Götaland (n = 18), Skåne (n = 6) and Halland (n = 8) (Figure 4). 

The number of infections caused by *V. cholerae* (non-O1/O139) was the highest of all identified *Vibrio* species (n = 183). Non-toxigenic *V. cholerae* infections were reported in 16 counties of Sweden. *Vibrio alginolyticus* caused 72 infections, with higher numbers in higher salinity counties of Sweden. The first vibriosis cases caused by *V. vulnificus* were observed in 2018 (n = 16), and since then, it has caused at least one infection per year. Over half of *V. vulnificus* infections have been reported in the Skåne (n = 13) county. The remaining infections were recorded at the *Vibrio* genus level and made up to 289 vibriosis cases over 18 years.

In the period of 2004–21, the mean summer season surface temperature on the Swedish coast of the Baltic Sea varied from 14.5 ± 1.3 °C on the coast of Gävleborg to 17.9 °C ± 1.4 on the coast of Uppsala County. There were significant differences in temperature among the Swedish counties (p < 0.05; Figure 4) and the years (p < 0.05; Figure 3). The total number of *Vibrio* spp. (n = 143), *V. alginolyticus* (n = 143) and *V. parahaemolyticus* (n = 143) infections significantly positively correlated with average SST (r_S_ = 0.39, r_S_ = 0.22, r_S_ = 0.27, respectively; p < 0.05).

In 2004–21, the mean summer season salinity on the Swedish coast of the Baltic Sea varied from 2.0 ± 0.1 PSU at the most northern Norrbotten County to 22.1 ± 1.7 PSU at the coast of Västra Götaland, which is the county closest to the straights of Kattegat and Skagerrak by Denmark, connecting the Baltic Sea to the North Sea. The total number of *Vibrio* spp., and *V. alginolyticus* and *V. parahaemolyticus* infections positively correlated with salinity (r_S_ = 0.39, r_S_ = 0.18 and r_S_ = 0.31, respectively; p < 0.05; n = 137). Meanwhile, the number of infections of non-toxigenic *V. cholerae* correlated negatively with salinity (r_S_ = -0.18).

### Prediction of *Vibrio* cases

#### A prognostic model calibration and internal validation based on *Vibrio* cases in Sweden

**Figure 5:**
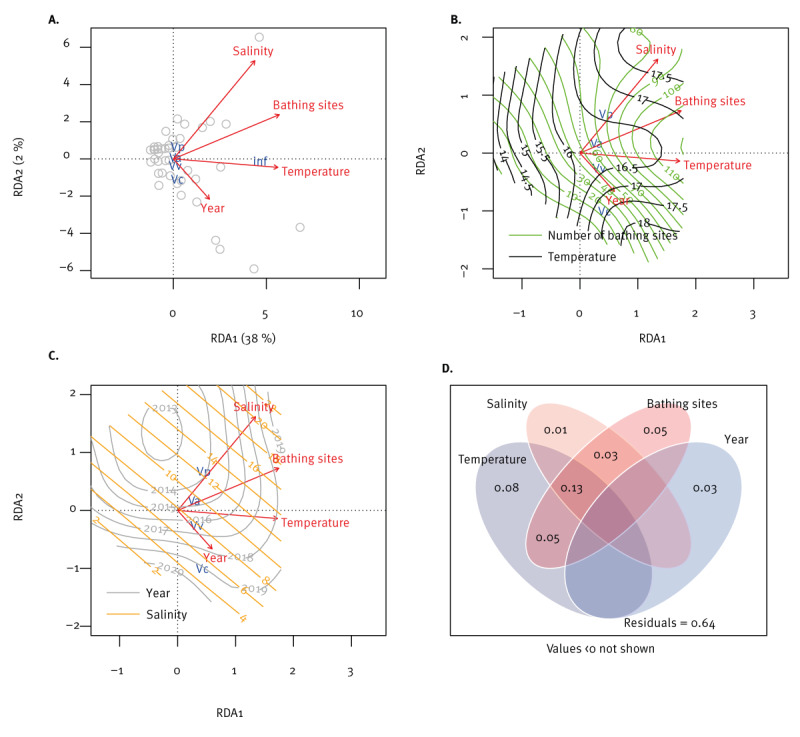
Redundancy analysis of vibriosis cases in Sweden, 2014–2021 (n = 14 counties)

We used the most comprehensive *Vibrio* dataset from Sweden to create a *Vibrio* infection prognostic model. Results of the RDA showed that the predictors explained 36% of *Vibrio* infection variation (Figure 5). The most significant explanatory variable was SST (F = 7.17, p < 0.01), followed by the number of bathing sites (F = 5.33, p = 0.01) and year (F = 4.40, p = 0.04). Total *Vibrio* infections correlated with temperature and number of designated bathing sites, *V. cholerae* (non-O1/O139) correlated with year and negatively correlated with salinity. *Vibrio vulnificus* partly correlated with temperature and year, while *V. alginolyticus* partly correlated with temperature and the number of designated bathing sites.

Inf: total number of cases; Va: *V. alginolyticus*; Vc: *V. cholerae* non-O1/O139; Vp: *V. parahaemolyticus;* Vv: *V. vulnificus.*

RDA (with scaling = 2) triplot (A) with the response variables (blue), predictors (red), and samples (dots); RDA biplots (B and C) with superimposed isolines of predictors; the relative importance of predictors based on the variance partitioning (D).

Response variables: total infections of *Vibrio* (inf), *V. alginolyticus* (Va), *V. cholerae* (Vc), *V. parahaemolyticus* (Vp), and *V. vulnificus* (Vv). Predictor variables: temperature, salinity, the number of designated coastal beaches and year.

#### External model validation based on *Vibrio* cases in Germany 

The accuracy of the internal validation of the model was relatively high (MAE ≤ 4.0), whereas the external validation was less accurate (MAE < 15.0). The validation of the model showed that, in different years for different Swedish counties, 0 to 16 of total infections were predicted; 0 to 2 infections for *V. alginolyticus* and *V. vulnificus*, 0 to 4 for *V. cholerae* (non-O1/O139) and 0 to 3 for *V. parahaemolyticus*. For Germany (external validation), an overestimation was determined for the total infections and *V. cholerae*, while the infections by *V. parahaemolyticus* and *V. vulnificus* were underestimated. The internal and external validations of the RDA are provided in Supplementary Figure S4.

#### Prediction of vibriosis cases for the Baltic Sea region countries

**Figure 6:**
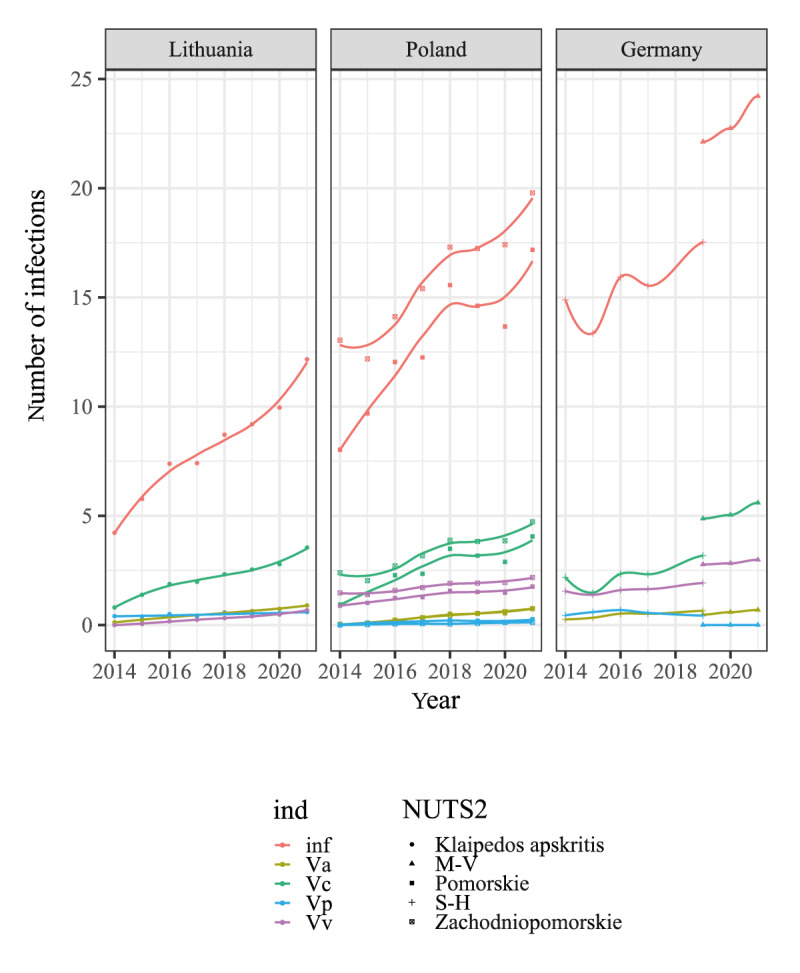
Predicted number of total *Vibrio* infections and specific species in Germany (n = 2 regions) 2014–2018, Lithuania (n = 1 region) and Poland (n = 2 regions), 2014–2021

We applied the model to predict infections caused by *Vibrio* from 2014 to 2021 in Germany, Lithuania and Poland. We found that there were some differences between the countries (Figure 6) but observed a common trend that infection numbers were predicted to increase over the years. The highest number of total cases was predicted in Germany, reaching up to 24 cases per region in 2021, while in Poland, the model predicted up to 20 infections per region bordering the BSR; in Lithuania, the prediction was up to 12 infections per county bordering the BSR. From the analysed species, the highest numbers of infections were predicted to be caused by *V. cholerae* (non-O1/O139) in all countries, and the secondary cause of infection by *V. vulnificus* in the case of Germany (from 2 to 3 per region per year) and Poland (from 1 to 2 cases per region per year).

Inf: total number of cases; M-V: Mecklenburg-Vorpommern; S-H: Schleswig-Holstein German federal states; Va: *V. alginolyticus*; Vc: *V. cholerae* non-O1/O139; Vp: *V. parahaemolyticus;* Vv: *V. vulnificus.*

Predictions by the RDA model in Germany for S-H (period 2014–18) and M-V regions (period 2018–21) (NUTS 2), Lithuania, Klaipedos apskritis (NUTS 3) and Poland for Pamorskie and Zachodniopamorskie regions (NUTS 2) during the period of 2014–21.The smooth lines were obtained by fitting local polynomial regressions with a span of 0.8.

## Discussion

Our study provides a comprehensive summary of *Vibrio* spp. and vibriosis cases around the BSR from 1994 to 2021. To our knowledge, this comprises the most extensive dataset available to date, encompassing a total of 1,553 vibriosis cases. Additionally, we compiled data on *Vibrio* spp. occurrence in the BSR waters in six countries. Our study primarily concerns the ecological aspect rather than an in-depth epidemiological analysis. Furthermore, it explores the predictive aspects of vibriosis in countries where *Vibrio* is present, yet surveillance of infections is limited.

One notable finding from our research is the exponential rise in recorded *Vibrio* cases across the BSR over the studied period. This trend is also noticeable within Sweden's epidemiological data (R^2^ = 0.43). In California, US, the *Vibrio* incidence rate increased from 0.4 (n = 154 cases) in 2013 to 0.7 per 100,000 (n = 277 cases) in 2019, with the highest incidence rate observed in 2018 at 0.9 per 100,000 (n = 338 cases) [13]. In 2018, during a notably warm summer marked by elevated temperatures reaching ca 25 °C on the Baltic coast of Lithuania [10], a maximum of 163 infections were reported in the BSR. Our study does not show a significant correlation between the total number of infections in the BSR and mean summer SST, possibly because the SST did not differ significantly over the years. In contrast, previously published studies used the maximum annual SST for the correlation with *Vibrio* incidence analysis and showed positive correlations [8,17]. However, in our study, a positive correlation between the number of infections caused by certain *Vibrio* species and the mean summer SST has been discerned in at the county level in Sweden.

The Baltic Sea is one of the fastest warming ecosystems [23] and the influence of global warming and elevated temperature on vibriosis incidence rates [8,17,24,25], and the abundance of *Vibrio* spp. [7,26] has been the subject of extensive deliberation. Salinity emerges as another critical factor driving *Vibrio* spp. distribution and abundance as substantiated by previous investigations [1,27]. We observed a significant negative relationship between the total number of infections reported in the BSR and salinity. However, this could have been affected by the unidentified species (n = 312) that have preferred lower salinity conditions. These unclassified infections may be either one of the ‘big four’ species or other globally recognised human infection-causing species such as *V. anguillarum* [28], *V. furnissii* [29], *V. harveyi* [16], *V. metschnikovii* [30], and *V. cincinnatiensis* [31]. In the BSR, infections have also been associated with *V. fluvialis* [32] and *V. navarrensis* [33].

Long-term monitoring data of *V. vulnificus* showed a positive correlation with SST in several bathing sites along the Baltic Sea coast of Schleswig-Holstein and Mecklenburg-Vorpommern in Germany. Our study revealed the absence of *V. vulnificus* in regions with salinity levels lower than 5.0 PSU, such as the Gulf of Bothnia, and higher than 17 PSU. In Sweden, counties located along higher average salinity waters, such as Halland and Västra Götaland (average salinity > 16.0 PSU), reported minimal infections attributed to *V. vulnificus*, ranging from 0 to 2 infections, as opposed to Skåne (n = 13) county with an average salinity of ca 12 PSU. Other studies identified a salinity range of 6.0–15.4 PSU favourable for *V. vulnificus* growth [17].

Conversely, *V. alginolyticus* exhibits a broader salinity range for growth, detected in 93% of all investigated bathing sites in Estonia; however, it has yet to be found on the coast of Lithuania. Hounmanou et al. [19] posit that *V. alginolyticus* has been responsible for over 50% of all reported cases in Denmark in less than a decade. Similar salinity and temperature conditions have been observed for *V. parahaemolyticus* and *V. cholerae* (non-O1/O139).

In addition to SST and SSS, various other parameters influence the distribution and abundance of *Vibrio* spp. Recent studies have revealed a positive correlation between *Vibrio* spp. and parameters associated with eutrophication, encompassing chlorophyll a and blue-green algae concentrations [10,27,34], dissolved organic matter, and coloured dissolved organic matter resulting from phytoplankton blooms [6,35]. Research by Riedinger et al. [36] suggests that mitigating nutrient inputs to curtail algal bloom events, which proliferate *V. vulnificus* growth, could potentially reduce risks to public health. Nonetheless, given the established correlation with chlorophyll a, regions classified as high-risk for eutrophication, such as the Gulf of Riga and the Gdansk Basin, according to Njock et al. [37], would similarly be deemed high risk for *Vibrio* infections.

However, it is imperative to note that despite the heightened risk, three south-eastern Baltic Sea countries, namely Latvia, Lithuania and Poland, have yet to institute comprehensive surveillance systems and monitoring programmes explicitly targeting *Vibrio* bacteria in bathing waters along their respective coastal areas. While all the BSR countries monitor *V. cholerae* serogroups O139 and O1, which are linked to cholera epidemics, the absence of surveillance for other *Vibrio* species thriving in the Baltic Sea hampers the ability to evaluate the prevalence and potential risks of *Vibrio* infections in these areas.

To estimate the number of overlooked infections in countries without surveillance, we developed a statistical model calibrated by the data from Sweden. The model considered environmental factors such as temperature and salinity as primary drivers of *Vibrio* infections. We also incorporated the number of designated bathing waters in coastal areas, as more bathing sites may lead to a greater number of visitors, increasing the risk of *Vibrio* exposure. The external validation of the model with data from Germany revealed an overestimation for the total infections and for *V. cholerae*, while the infections by *V. parahaemolyticus* and *V. vulnificus* were underestimated. According to the model, in 2021, we anticipated up to 20 infection cases per region in Poland and up to 12 cases in Lithuania. However, the officially reported cases are much fewer – only eight cases in total have been documented in Poland in 2018–21 [18,38,39], and none have been noted in Lithuania. This suggests a potential underreporting of *Vibrio* infections in these regions. 

Because of exceptionally high SST in the summer of 2018, a *Vibrio* warning was disseminated for the bathing sites along the Lithuanian Baltic Sea coast, based on the ECDC *Vibrio* Map Viewer [1]. In response, subsequent assessments of bathing waters for *Vibrio* presence by local beach managers yielded negative results, contrasting with the results of the study by Gyraite et al. [10]. This discrepancy highlights the crucial role of the effort and knowledge in monitoring bathing water quality. Estonia serves as a case study in this regard, where surveillance and monitoring programmes were established in 2019, reporting nine infection cases and monitoring *Vibrio* spp. in 15 bathing sites within 2 years. Furthermore, the exponential increase in *Vibrio* infections in the BSR and countries like Sweden may indicate enhanced, improved species identification methods and equipment and skilled personnel adept in disease diagnostics. Our analysis revealed that only annual data were available in certain countries, which lacked information on the specific locations and timing of *Vibrio* infections. It is crucial to have detailed data on where and when infections occur for effective surveillance, outbreak response, environmental monitoring and public health interventions. Public awareness, too, contributes to these efforts. Personal communication with project stakeholders, the general public, scientists from Poland and Latvia, and representatives of major hospitals in Lithuania revealed a marked lack of awareness regarding non-notifiable agents such as *Vibrio* spp. Nonetheless, this knowledge gap does not imply the absence of *Vibrio* infections, particularly when environmental conditions align with those in neighbouring countries where infections have been documented.

Our study has several limitations that need to be considered. Firstly, the data sources varied notably; with some data provided directly by the national authorities, and other data extracted from literature, which often lacked clarity. For example, cases without travel history were considered as non-travel-related; a place of residency was used as a proxy when the site of infection was not identified, etc. Secondly, the accuracy of the data was variable. Some countries provided highly detailed information at the NUTS 3 level or even a specific bathing site where infection might have been obtained, while for others, we could only acquire data at the NUTS 1 level. Moreover, countries such as Germany and Sweden provided data spanning 27 and 17 years, respectively, in comparison to Estonia, which contributed only 2 years of surveillance data. Thirdly, the precision of infection dates varied. In some cases, exact dates were available, but in the majority of cases, only the year was recorded. Finally, for our prognostic model, environmental data such as temperature and salinity were not always available in the official data sets provided by the authorities. Consequently, we had to extract this information from general databases, which may impact the precision and validity of our environmental parameters, potentially influencing the accuracy of our predictive model. 

## Conclusions

This study provides a comprehensive and, to our knowledge, the largest dataset of *Vibrio* spp. infections in the BSR from 1994 to 2021. It shows an exponential increase in recorded cases of infection in the BSR region and a broad prevalence of *Vibrio* spp. throughout the BSR. The predictions of the statistical model showed a potential underreporting of *Vibrio* infections in countries without surveillance, highlighting the need for an adequate system of infection surveillance and monitoring in the BSR and across Europe. In addition, projections of climate change and environmental suitability for *Vibrio* identified the BSR as a hotspot for *Vibrio* infections. Given the incomplete epidemiological data and fragmented research efforts, this comprehensive review calls for international, transdisciplinary and multisectoral collaboration to develop effective transnational management of these pathogens. In our opinion, the first step towards a better system could be to make these *Vibrio* spp. notifiable in Europe so that there is a legal reporting mechanism, followed by the establishment of an international framework led by an overarching international organisation, e.g. an international reference laboratory, for the exchange of epidemiological, clinical and environmental information.

## Supplementary Data

705000 Supplement
