# Supplementary material for: Epidemiological and environmental investigation of the ‘big four’ Vibrio species, 1994 to 2021: a Baltic Sea retrospective study
Source: Euro Surveill. 2024 Aug 8;29(32):2400075. doi: 10.2807/1560-7917.ES.2024.29.32.2400075 (PMC11312017; doi:10.2807/1560-7917.ES.2024.29.32.2400075)
Supplement: Supplement [file 24-00075_GYRAITE_SUPPLEMENT.pdf]

SUPPLEMENTARY MATERIAL

This supplementary material is hosted by *Eurosurveillance* as supporting information alongside the article "Epidemiological and environmental investigation of the 'big four' *Vibrio* species, 1994 to 2021: a Baltic Sea retrospective study" on behalf of the authors who remain responsible for the accuracy and appropriateness of the content. The same standards for ethics, copyright, attributions and permissions as for the article apply. Eurosurveillance is not responsible for the maintenance of any links or email addresses provided therein.

Table S1. *Survey of case surveillance data on Vibrio spp.*

Information about the project BaltVib

BaltVib: Pathogenic *Vibrio* bacteria in the current and future Baltic Sea waters	
**Lead Partner**	The Leibniz Institute for Baltic Sea Research Warnemünde (**Germany**);
**Project Partners**	GEOMAR Helmholtz Centre for Ocean Research Kiel (**Germany**); the Marine Research Institute of the Klaipėda university (**Lithuania**); the University of Copenhagen (**Denmark**); the Estonian University of Life Sciences, the Royal Institute of Technology Stockholm (**Sweden**); the Finnish Åbo Akademi University (**Finland**) and the National Research Institute of Marine Fisheries (**Poland**).
**Funding**	2019-2020 BiodivERsA joint call for research proposals, under the BiodivClim ERA-Net COFUND programme
**Project scientific contact**	
**The database contact**	

Information about the data provider

NAME OF INSTITUTION	CONTACT PERSON	PHONE NUMBER	EMAIL ADDRESS	POSITION

Information about the infection cases

#	COUNTRY	DATE	BATHING/INFECTION SITE	BATHING_ID	SEX (F/M)	AGE	LETHAL (YES/NO)	*Vibrio* spp.	Additional information (symptoms; risk factor; activity)	Comments
i.e.	*Germany*	*1994 June 17*	*Usedom*	*DEMV_PR_1_0707*	*F*	*73*	*NO*	*V. vulnificus*	*Person with liver dysfunction; infection after bathing*	

Table S2. *Survey of Vibrio spp. environmental monitoring data*

Information about the project BaltVib

BaltVib: Pathogenic *Vibrio* bacteria in the current and future Baltic Sea waters	
**Lead Partner**	The Leibniz Institute for Baltic Sea Research Warnemünde (**Germany**);
**Project Partners**	GEOMAR Helmholtz Centre for Ocean Research Kiel (**Germany**); the Marine Research Institute of the Klaipėda university (**Lithuania**); the University of Copenhagen (**Denmark**); the Estonian University of Life Sciences, the Royal Institute of Technology Stockholm (**Sweden**); the Finnish Åbo Akademi University (**Finland**) and the National Research Institute of Marine Fisheries (**Poland**).
**Funding**	2019-2020 BiodivERsA joint call for research proposals, under the BiodivClim ERA-Net COFUND programme
**Project scientific contact**	
**The database contact**	

Information about the data provider

NAME OF INSTITUTION	CONTACT PERSON	PHONE NUMBER	EMAIL ADDRESS	POSITION

Information about the infection cases

#	Country	Date	Monitoring site *(State, municipality, bathing site, coordinates)*	Coordinates	*Vibrio* spp. monitored *(V. vulnificus, V. parahaemolyticus, V. cholerae)*	The assement method *(Cultivation/molecular)*	The quantity *(MPN/gene copies/CFU/presence /absence)*	Water temperature, °C	Water salinity, PSU	Comments *(i.e. source of info, etc.)*
i.e.	*Germany*	*2017 Aug 10*	*MV, Karlshagen, bathing site ID 703*		*V. vulnificus*	*Cultivation*	*10.000 CFU/L*	*22.2*	*7.6*	

Table S3. *The official authority data used for the analysis of Vibrio spp. infections*

Country/ County	Infection surveillance				
	**Number of infection cases**	**Year of surveillance**	**Site where infection happened**	**Date when infection happened**	**Data resources**
**Germany** Schleswig-Holstein	8	2003-2021	at NUTS3 level	For 5 cases exact date (yyyy-mm-dd) is known.	Infection surveillance data provided by Landesamt für Gesundheit und Soziales (LAGuS S-H)<br>SST data of an exact date and county extracted from https://satbaltyk.iopan.gda.pl<br>SSS data of an exact date and county extracted from https://satbaltyk.iopan.gda.pl
**Germany** Mecklenburg-Vorpommern	49	1994-2018	exact bathing site known for 43 cases	Only year known	Infection surveillance data provided by Landesamt für Gesundheit und Soziales (LAGuS M-V).<br>The average summer SST and SSS for a specific bathing site were extracted from the provided official monitoring data; and from the Oceanographic Database Search with Interactive Navigation (ODIN 2) provided and maintained by Leibniz Institute for the Baltic Sea Research, Warnemuende (IOW) (https://odin2.io-warnemuende.de/).<br>Data from the upper 10 meters were extracted from the Obboje station (14.1497; 54.0844).
**Estonia**	9	2020-2021	NUTS3 level	Exact date	Infection surveillance data provided by the Health Board of Estonia.<br>SST data of an exact date and county extracted from https://satbaltyk.iopan.gda.pl<br>SSS data of an exact date and county extracted from https://satbaltyk.iopan.gda.pl
**Sweden**	625	2004-2021	at NUTS3 level	Only year known	Infection surveillance data provided by the Public Health Agency of Sweden.<br>The average summer (June-September) STT and SSS were extracted from the database (SHARKweb), hosted by the Swedish Meteorological and Hydrological Institute (https://sharkweb.smhi.se/hamta-data/).<br>The upper 10 meters were extracted from the territorial waters of each County allocated on the Baltic Sea coast.
**Finland**	221	1995-2021	Country-level	Only year known	*V. cholerae* infection surveillance data provided by The Finnish Institute for Health and Welfare; was not included in the analysis due to the lack of infection origin.

Table S4. *The official authority data used for the analysis of Vibrio spp. monitoring in the Baltic Sea waters*

Country/ County	Monitoring in the water			
	**Monitored Vibrio parameter**	**Year of monitoring**	**Temperature of water**	**Salinity of water**
**Germany** Schleswig-Holstein	Abundance of *V. cholerae*, *V. vulnificus*, *V. alginolyticus*, and *V. parahaemolyticus*	2014-2016, 2018-2019, and 2021	*Vibrio* monitoring is provided by Infection surveillance data provided by Landesamt für Gesundheit und Soziales (LAGuS M-V & S-H).<br>SST was measured simultaneously during monitoring.<br>SSS is provided only for the M-V region.	
**Germany** Mecklenburg-Vorpommern	2004-2008 presence/absence indications of the 'big four' *Vibrio* species<br>2008-2021 *V. vulnificus* quantification data and the presence/absence of *Vibrio* spp.	2004-2021	*Vibrio* monitoring and temperature data provided by Landesamt für Gesundheit und Soziales (LAGuS M-V), Department of Health.<br>SST and SSS were measured simultaneously during monitoring (2004 – 2021).	
**Estonia**	Presence of four *Vibrio* species	2019-2021	*Vibrio* monitoring and temperature data provided by the Health Board of Estonia.	
**Sweden**	-	-	-	-
**Finland**	Presence/absence and quantity of four *Vibrio* species	2021	Simultaneously during monitoring	No data

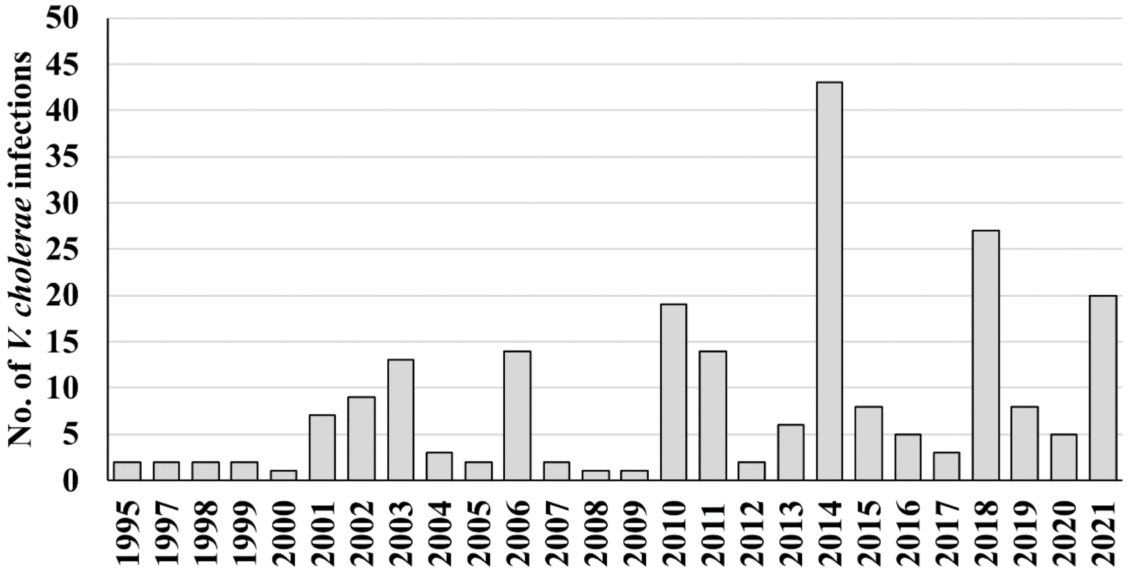

Figure S1. *V. cholerae reported cases in Finland, 1995 – 2021*

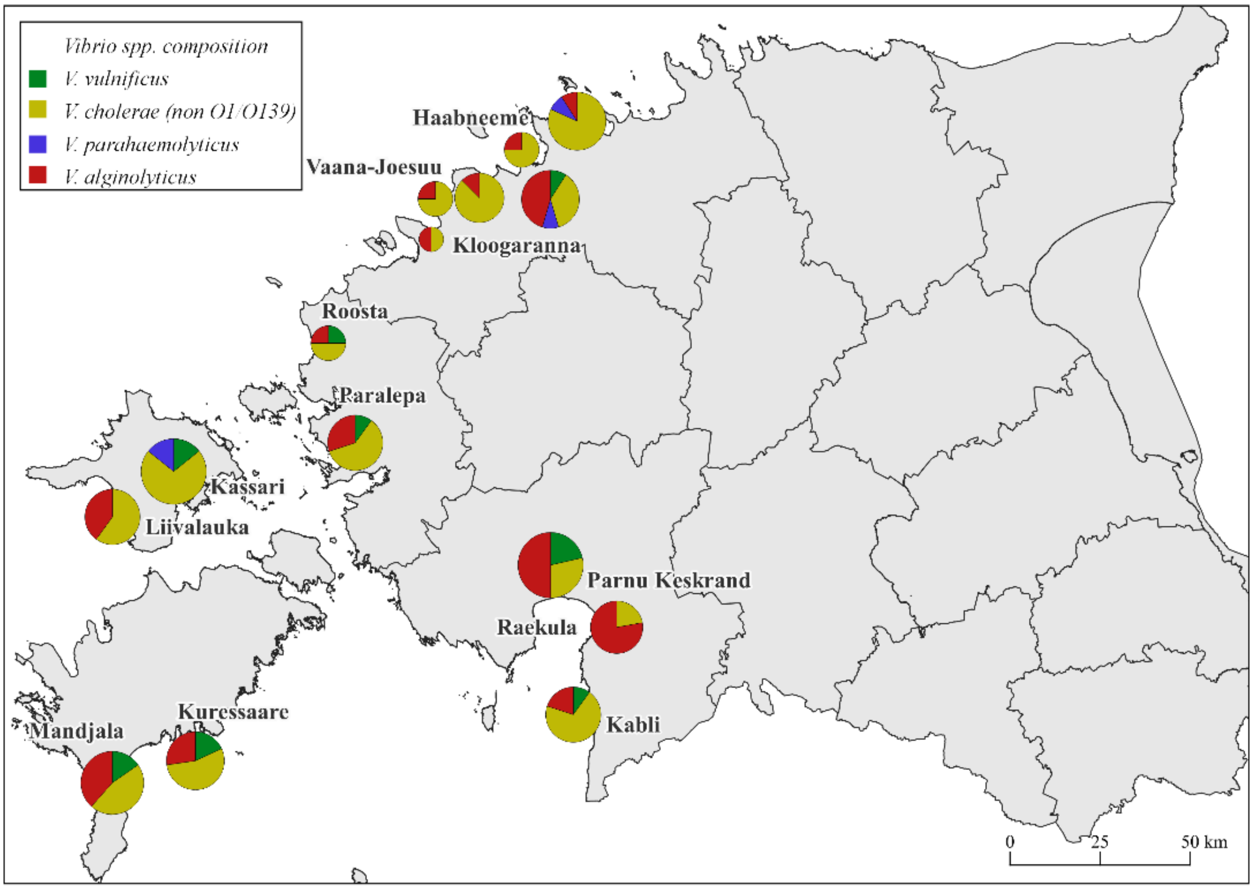

Figure S2. *Vibrio spp. diversity observed in the Estonian Baltic Sea waters in 2019 – 2021*

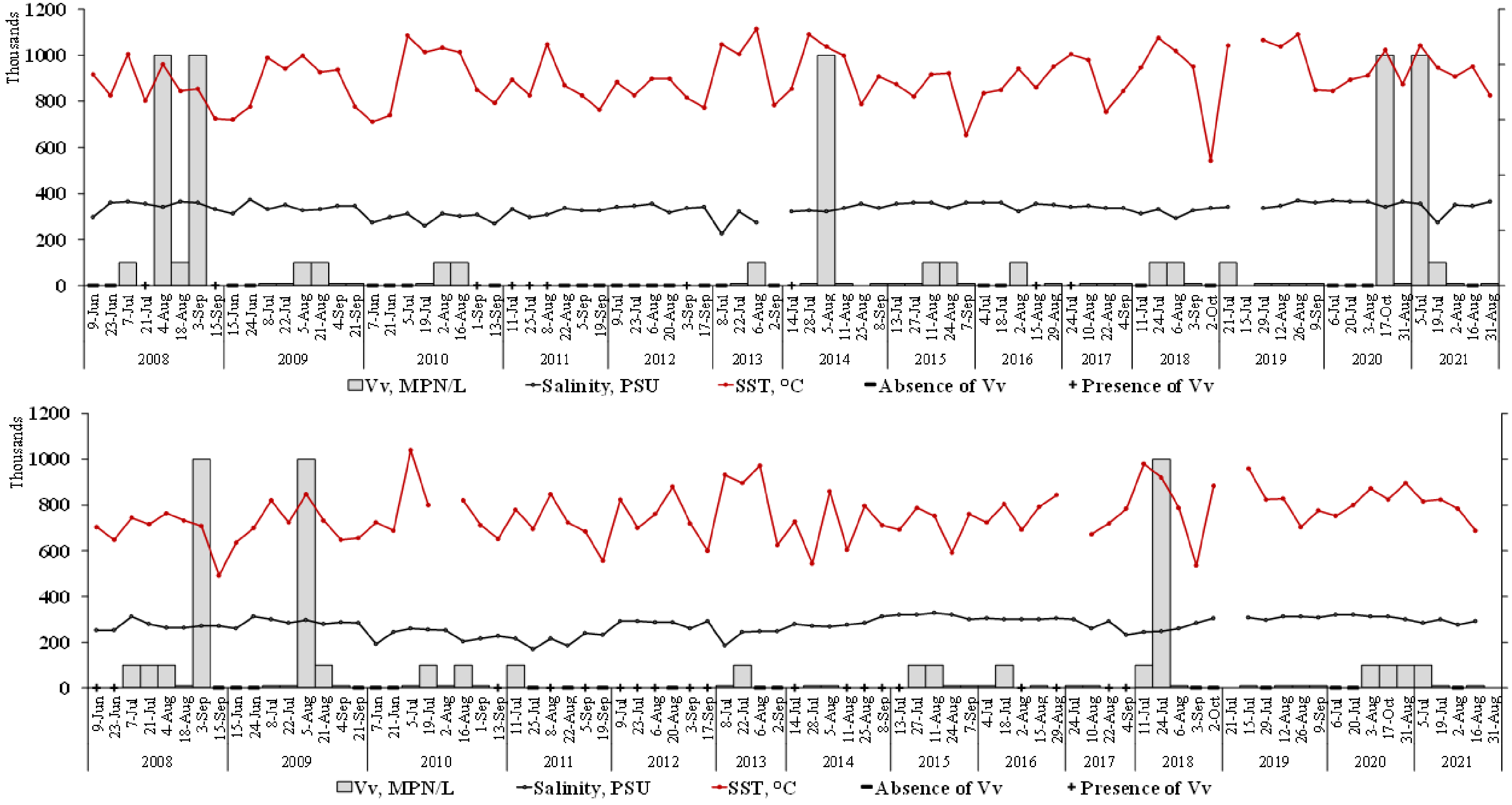

Figure S3. *V. vulnificus monitoring data at Karlshagen (upper graph) and Lubmin (lower graph) bathing sites in 2008 – 2021. +/- on the x axis represents presence and absence, bar plot - the abundance, red line – SST, black line – SSS.*

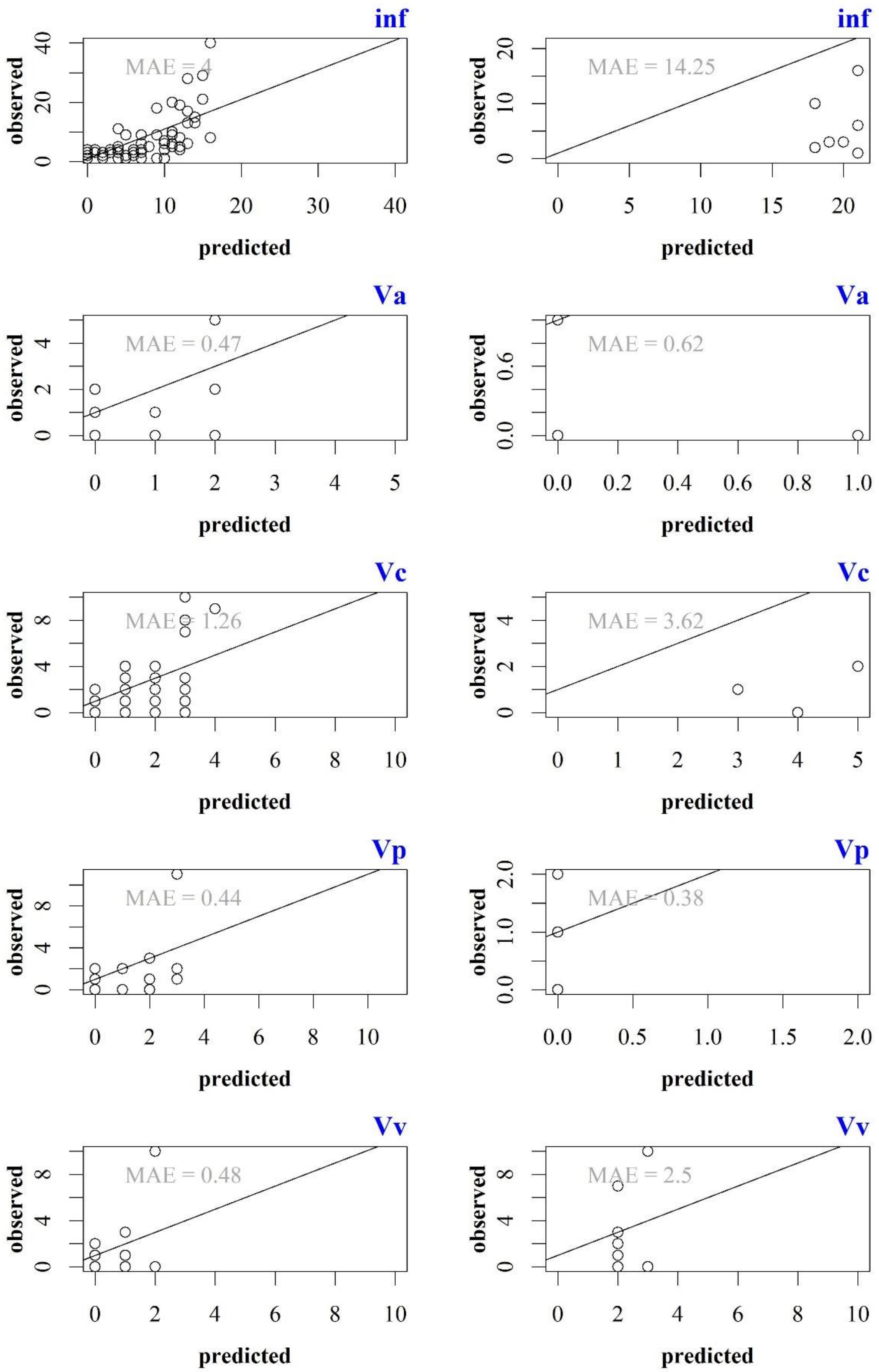

Figure S4. *Internal (left side) and external (right side) validations of the RDA predictions for each response variable (blue): the total infections of Vibrio (inf) and specific species (Va – V. alginolyticus, Vc – V. cholerae non-O1/O139, Vp – V. parahaemolyticus, and Vv – V. vulnificus). Mean absolute error (MAE) is provided in plots*
